# ^18^F-FDG PET/CT-based recurrence patterns and survival outcomes in esophageal carcinoma: a single center experience

**DOI:** 10.2478/raon-2026-0047

**Published:** 2026-09-07

**Authors:** Milica Kotur, Strahinja Odalovic, Jelena Petrovic, Nikola Pantic, Isidora Grozdic Milojevic, Dragana Sobic Saranovic, Predrag Sabljak, Vera Artiko

**Affiliations:** 1 Center for Nuclear Medicine with PET, University Clinical Center of Serbia, Belgrade, Serbia; 2 Faculty of Medicine, University of Belgrade, Belgrade, Serbia; 3 Clinic for Digestive Surgery, University Clinical Center of Serbia, Belgrade, Serbia

**Keywords:** recurrence of esophageal carcinoma, ^18^F-FDG PET/CT, semiquantitative and quantitative ^18^F-FDG PET/CT parameters, progression-free survival, overall survival

## Abstract

**Background:**

This study aimed to evaluate differences among three patient groups with suspected recurrent esophageal carcinoma, defined by ^18^F-fluorodeoxyglucose positron emission tomography/computed tomography (^18^F-FDG PET/CT) findings, in quantitative and semiquantitative PET/CT parameters and serum tumor markers, and to assess their impact on progression-free survival (PFS) and overall survival (OS).

**Patients and methods:**

This retrospective cohort study included 77 patients with positive ^18^F-FDG PET/CT findings between January 2014 and December 2025. Based on ^18^F-FDG PET/CT findings, patients were classified into three groups: G1 (isolated locoregional recurrence), G2 (isolated metastases), and G3 (both locoregional disease and metastases). Semiquantitative and quantitative ^18^F-FDG PET/CT parameters, histopathological characteristics, and blood tumor marker levels were compared among the groups. For survival analysis, patients were stratified into low and high groups according to the median value of each parameter.

**Results:**

Elevated carcinoembryonic antigen (CEA) levels were significantly more frequent in G2 and G3 compared with G1 (p = 0.005), while no significant difference was observed for carbohydrate antigen 19-9 (CA19-9) levels (p = 0.280). Significant differences in all analyzed ^18^F-FDG PET/CT parameters were found among the three groups, with the highest values in G3. Patients with lower maximal standardized uptake values (SUVmax) and SUVmean values demonstrated significantly longer PFS and OS. Lower metabolic tumor volume (MTV) and total lesion glycolysis (TLG) values were associated with significantly longer progression-free survival (PFS), while overall survival (OS) was also longer, although without statistical significance.

**Conclusions:**

Studies stratifying patients by isolated locoregional recurrence, isolated metastases, and combined locoregional/metastases on ^18^F-FDG PET/CT remain limited. Our findings suggest that disease spread patterns and semiquantitative/quantitative ^18^F-FDG PET/CT parameters may provide important prognostic value in patients with esophageal carcinoma.

## Introduction

Esophageal carcinoma (EC) is one of the leading causes of cancer mortality worldwide.^1^ The two main histopathological subtypes of this malignancy are esophageal squamous cell carcinoma (ESCC) and esophageal adenocarcinoma (EAC).^1,2^ The distribution of these subtypes shows marked geographical variation, with EAC being more prevalent in Europe and North America, while ESCC predominates in Asia and Africa.^3^

The treatment of esophageal carcinoma depends on histopathological subtype, tumor location, disease stage, and the patient’s overall clinical condition and includes surgery, chemotherapy (CHT), and radiotherapy (RT). Despite advances in therapeutic strategies, the prognosis remains poor, primarily due to the high rate of disease recurrence.^4^ After initial treatment, early detection of disease recurrence, accurate localization, and timely initiation of therapy are crucial for disease course and patient outcome.^5,6^

Different studies have classified esophageal carcinoma recurrence in different ways. Different approaches have been proposed, with relapse most commonly categorized as: (1) local, (2) regional, and (3) metastases.^7^ In addition, some studies have classified esophageal carcinoma relapse based on ^18^F-FDG PET/CT findings, distinguishing between local recurrence and metastatic disease, as well as more complex categories, including isolated locoregional disease, locoregional disease associated with lymph node metastases (without distant metastases), and disease with simultaneous lymph node and distant metastases. In general, regardless of how esophageal carcinoma recurrence is classified, patients with metastases have worse progression free survival (PFS) and overall survival (OS) than those with isolated locoregional recurrence.^7-9^

Although previous studies have highlighted the high sensitivity of ^18^F-FDG PET/CT in detecting recurrent disease^10^, there is still a clear gap in understanding the prognostic value of quantitative and semiquantitative ^18^F-FDG PET/CT parameters, including standardized uptake values (SUVmax, SUVpeak, and SUVmean), metabolic tumor volume (MTV), and total lesion glycolysis (TLG), across different recurrence patterns identified by ^18^F-FDG PET/CT isolated locoregional recurrence, metastatic relapse, and combined locoregional and metastatic relapse. Furthermore, these parameters have not been systematically evaluated and compared among these three groups.

Therefore, the aim of this study was to evaluate differences among three specific patient groups, defined solely on the basis of ^18^F-FDG PET/CT findings, with regard to key quantitative and semiquantitative ^18^F-FDG PET/CT parameters, as well as to assess their impact on PFS and OS.

## Patients and methods

### Patients

This retrospective cohort study included 77 patients with suspected relapse of esophageal carcinoma and positive ^18^F-FDG PET/CT findings between January 2014 and December 2025. Inclusion criteria were: (1) patients with histologically confirmed primary esophageal carcinoma; (2) positive ^18^F-FDG PET/CT findings; (3) available data on histopathological type; (4) previously received therapy (surgical resection, CHT, and/or RT); (5) a minimum interval of three months following surgery or RT, or four weeks after the last cycle of CHT; and (6) available values of tumor markers carcinoembryonic antigen (CEA) and CA 19-9 obtained within one month prior to the ^18^F-FDG PET/CT scan. Exclusion criteria were: (1) negative ^18^F-FDG PET/CT findings; (2) glycemia above 11 mmol/L; and (3) history of another malignancy. ^18^F-FDG PET/CT was performed due to suspected locoregional recurrence and/or metastatic disease.

Medical data collected included demographic characteristics (age and sex), tumor characteristics (histopathological type), and details of prior treatment (surgery, CHT, and/or RT). Serum tumor marker levels (CEA and CA 19-9), measured within one month prior to the ^18^F-FDG PET/CT examination, were also recorded.

Clinical and imaging follow-ups for at least one year after the ^18^F-FDG PET/CT examination were used as a gold standard for assessment of the progression of the disease.

Written informed consent was obtained from all participants. The study was approved by the Institutional Ethics Committee (approval No. 1500/45).

### ^18^F-FDG PET/CT protocol, acquisition, and interpretation

All patients fasted for at least six hours prior to ^18^F-FDG PET/CT imaging (Biograph True64 hybrid scanner, Siemens). Blood glucose levels were measured before tracer administration, and only patients with values below 11 mmol/L proceeded with the examination. ^18^F-FDG was administered intravenously at a dose of 5.5 MBq/kg.

Image acquisition started 60 minutes after tracer injection. Initially, a low-dose, non-contrast-enhanced CT scan was performed (120 kV, 45 mAs, slice thickness 5 mm, pitch 1.5, rotation time 0.5 s), followed by a three-dimensional whole-body PET acquisition extending from the skull to the proximal femur (6–7 bed positions, 3 minutes per position). PET images were reconstructed using the ordered subset expectation maximization (OSEM) algorithm.

All images were analyzed on a dedicated workstation (syngo.via, version 2008B, Siemens Healthineers). The evaluation included both visual (qualitative) and semiquantitative/quantitative assessments. Any focal ^18^F-FDG uptake exceeding physiological distribution and not attributable to benign processes was interpreted as pathological. For each suspicious lesion, parameters including SUVmax, SUVpeak, SUVmean, MTV, and TLG were recorded.

Positive findings on ^18^F-FDG PET/CT were classified as either locoregional recurrence or metastatic disease. Locoregional lesions were defined as pathological ^18^F-FDG uptake within the surgical field and anastomosis in patients who underwent surgery or at the primary esophageal tumor site in non-operated patients. All other sites of pathological focal uptake were considered metastatic.

### Patient groups based on ^18^F-FDG PET/CT findings

Based on ^18^F-FDG PET/CT findings, patients were classified into three groups (G1–G3). Group G1 included patients with isolated locoregional disease on ^18^F-FDG PET/CT. Group G2 included patients with metastases without evidence of locoregional recurrence on ^18^F-FDG PET/CT. Group G3 included patients with both locoregional disease and metastases detected on ^18^F-FDG PET/CT. Semiquantitative and quantitative ^18^F-FDG PET/CT parameters—SUVmax, SUVmean, SUVpeak, MTV, and TLG values—were recorded for the locoregional lesion in the G1 group; the most metabolically active metastatic lesion in the G2 group; and separately for both the locoregional and the most metabolically active metastatic lesions in the G3 group. For comparisons of semiquantitative and quantitative ^18^F-FDG PET/CT parameters between groups, the higher value of each parameter obtained either from the locoregional lesion or from the most metabolically active metastatic lesion was selected for patients in the G3 group. These values were subsequently compared with the corresponding parameter values in the G1 and G2 groups.

### Patient groups based on the therapy administered before the ^18^F-FDG PET/CT examination

Patients were also divided in relation to the previously applied therapeutic modality into three groups: therapeutic group 1 (Th1), therapeutic group 2 (Th2) and therapeutic group 3 (Th3). The Th1 includes patients who underwent only surgical resection, without the use of CHT or RT. The therapeutic group Th2 consisted of patients who, in addition to surgical intervention, also underwent CHT and/or RT. The Th3 included patients who received CHT and/or RT, without previous surgical resection.

### Follow-up and survival analysis

Patients were followed for at least one year after the ^18^F-FDG PET/CT examination. During the follow-up period, disease progression was recorded. Progression is defined as the appearance of new lesions, an increase in the dimensions of existing lesions, or an increase in their metabolic activity, detected by conventional radiological methods (CT or magnetic resonance imaging) or on subsequent ^18^F-FDG PET/CT, as well as death due to the underlying disease or from other causes (based on data from medical records). PFS was calculated from the date of the ^18^F-FDG PET/CT examination to the date of confirmed disease progression, or until the end of the follow-up period if no progression was recorded. OS was defined as the time from the date of the ^18^F-FDG PET/CT examination to death from any cause or to the last follow-up. For survival analysis, patients were further stratified into two groups based on the median value of each ^18^F-FDG PET/CT parameter (low ≤ median and high > median).

### Statistical analysis

Statistical analysis was performed using EZR (Easy R) software, version 1.70, a graphical user interface for the R statistical programming language (version 4.5.2; The R Foundation for Statistical Computing, Vienna, Austria). Continuous variables were expressed as median and range, while categorical variables were presented as absolute numbers and percentages. Differences between two independent groups were assessed using the Mann-Whitney U test, whereas comparisons among three groups were performed using the Kruskal-Wallis test. When statistically significant, post-hoc pairwise comparisons were conducted using the Mann-Whitney U test with Holm correction for multiple testing. Categorical variables were compared using the Chi-square test or Fisher’s exact test, as appropriate. PFS and OS were analyzed using the Kaplan–Meier method, and differences between survival curves were assessed using the log-rank test. A p value < 0.05 was considered statistically significant.

## Results

### Patient characteristics and clinical findings

The study included 77 patients with previously histopathologically confirmed esophageal carcinoma and positive ^18^F-FDG PET/CT findings, performed due to suspected disease relapse after therapy. The mean age of the patients was 60.3 years (32–77). Of the total number of subjects, EAC was identified in 40 patients (51.9%), while 37 patients (48.1%) had ESCC. Detailed demographic characteristics, tumor marker levels, and data on previously applied therapeutic modalities according to group classification are presented in Table 1.

**TABLE 1.** Baseline demographic, clinical, and tumor characteristics of patients stratified by ^18^F-FDG PET/CT group classification

Parameter	G1	G2	G3	P
**N**	10	34	33	-
**Age** Med (range)	61.5 (46-76)	63.5 (35-78)	63.0 (32-73)	0.765
**Sex** Male/female	8/2	29/5	27/6	0.894
**Histopathology type EAC/ESCC**	9/1	12/22	19/14	**0.007**
**CEA** Normal/elevated	8/2	11/23	8/25	**0.005**
**CA19-9** Normal/elevated	5/5	12/22	8/25	0.280
**Therapy** Th1/Th2/Th3	6/2/2	10/17/7	4/16/13	0.030*F

1 ^18^F-FDG PET/CT = ^18^F-fluorodeoxyglucose positron emission tomography/computed tomography; CA19-9 = Carbohydrate antigen 19-9; CEA = carcinoembryonic antigen; EAC = esophageal adenocarcinoma; ESCC = esophageal squamous cell carcinoma;

* F = Fisher’s exact test; G1 = isolated locoregional disease; G2 = metastases without locoregional recurrence; G3 = locoregional disease and metastases; Th1 = surgery only; Th2 = surgery plus chemotherapy and/or radiotherapy; Th3 = chemotherapy and/or radiotherapy without prior surgery

1 P values were calculated using the Chi-square or Fisher’s exact test; age was analyzed using the Kruskal-Wallis test

**FIGURE 1.**
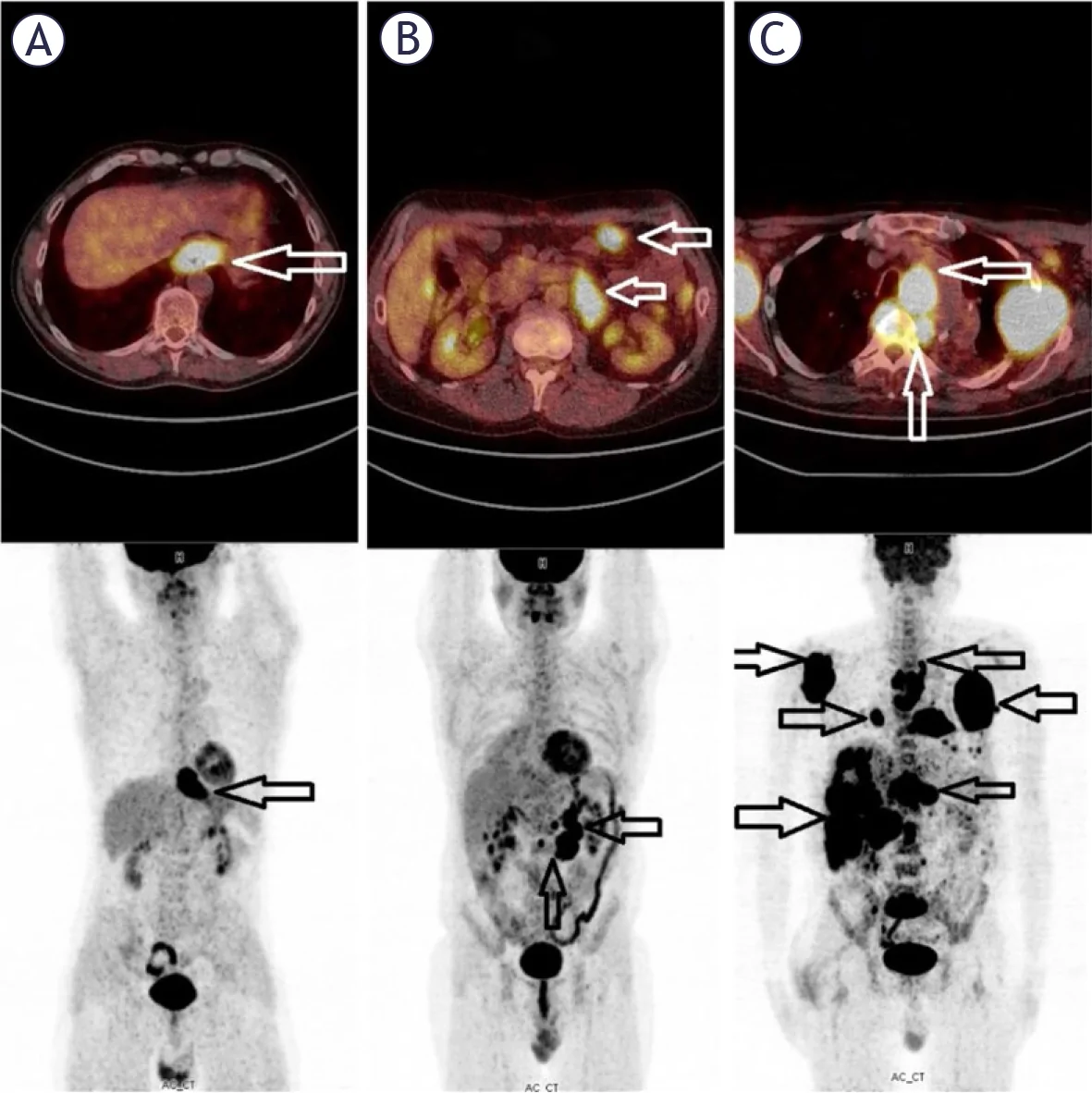
Representative imaging examples of patients from each study group (G1–G3). **(A)** Patient from the G1 group on 18F-FDG PET/CT demonstrating isolated locoregional esophageal recurrence. The white arrow indicates the locoregional lesion on the fused ^18^F-FDG PET/CT image, while the black arrow indicates the locoregional lesion on the MIP image. **(B)** Patient from the G2 group on ^18^F-FDG PET/CT demonstrating metastatic sites of esophageal recurrence without locoregional recurrence. The white arrows indicate metastatic lesions on the fused ^18^F-FDG PET/CT image, while the black arrows indicate metastatic lesions on the MIP image. **(C)** Patient from the G3 group on ^18^F-FDG PET/CT demonstrating metastatic sites of esophageal recurrence with locoregional recurrence. The white arrows indicate lesions on the fused ^18^F-FDG PET/CT images, while the black arrows indicate metastatic lesions on the MIP image.

A total of 10 patients were assigned to the G1 group, 34 to the G2 group, and 33 to the G3 group (representative cases from each group are illustrated in Figure 1). No statistically significant differences in age and sex were observed among the G1, G2, and G3 groups (p = 0.765 and p = 0.894, respectively). A statistically significant difference was found in histopathological type (p = 0.007), with EAC being more prevalent in the G1 group, while ESCC predominated in the G2 group.

Elevated CEA levels were significantly more frequent in the G2 and G3 groups compared to G1 (p = 0.005). In addition, a statistically significant difference in the applied therapeutic modalities was observed among the groups (p = 0.030). Although elevated blood carbohydrate antigen 19-9 (CA19-9) values were more common in G2 and G3 groups, this difference was not statistically significant (p = 0.280).

### Semiquantitative and quantitative ^18^F-FDG PET/CT analysis

Analysis of semiquantitative and quantitative ^18^F-FDG PET/CT parameters revealed statistically significant differences among the groups (G1-G3) for all examined parameters, with the highest level of significance observed for SUVmean (p < 0.001). The highest values of all analyzed parameters were recorded in the G3 group (Table 2).

**TABLE 2.** Semiquantitative and quantitative ^18^F-FDG PET/CT parameters according to group classification

Parameter	G1	G2	G3	P
**SUVmax**	5.9 (4.2-33.7)	7.8 (1.9-29.3)	12.7 (3.6-30.5)	**0.001**
**SUVpeak**	4.3 (3.1-30.1)	6.1 (1.3-24.7)	9.9 (3.3-23.2)	**0.001**
**SUVmean**	3.1 (2.2-21.5)	3.9 (1.6-14.9)	6.7 (2.3-20.3)	**<0.001**
**MTV**	8.4 (0.9-313.3)	13.2 (0.5-121.0)	24.4 (3.8-292.5)	**0.007**
**TLG**	9.7 (3.3-74.4)	19.5 (0.7-1768.5)	30.1 (2.9-790.5)	**0.042**

1 ^18^F-FDG PET/CT = ^18^F-fluorodeoxyglucose positron emission tomography/computed tomography; G1 = isolated locoregional disease; G2 = metastases without locoregional recurrence; G3 = locoregional disease and metastases; MTV = metabolic tumor volume; SUV = standardized uptake value; TLG = total lesion glycolysis.

1 Continuous variables are expressed as median (range). Differences between groups were assessed using the Kruskal-Wallis test. Post-hoc pairwise comparisons using the Mann–Whitney U test with Holm correction showed significant differences for SUVmax between G2 and G3 (p = 0.002), for SUVpeak between G2 and G3 (p = 0.002), for SUVmean between G1 and G3 (p = 0.008) and between G2 and G3 (p < 0.001), and for MTV between G2 and G3 (p = 0.007). Although TLG differed significantly in the Kruskal–Wallis test, post-hoc pairwise comparisons did not reach statistical significance.

Further analysis showed that ^18^F-FDG PET/CT parameters (SUVmax, SUVpeak, SUVmean, MTV, and TLG) did not differ significantly according to histopathological tumor type (EAC vs. ESCC; p > 0.05 for all parameters). Additionally, no statistically significant differences were observed in ^18^F-FDG PET/CT parameter values with respect to the applied therapeutic modalities (Th1, Th2, and Th3) (Table 3).

**TABLE 3.** ^18^F-FDG PET/CT metabolic parameters by histopathology and treatment groups

Parameter	EAC med (range)	ESCC med (range)	P	Th1 med (range)	Th2 med (range)	Th3 med (range)	P
**N**	40	37	-	20	35	22	-
**SUVmax**	10.2 (1.9-33.7)	9.9 (3.6-29.3)	1.000	11.2 (4.2-33.7)	7.6 (1.9-23.7)	11.9 (4.2-30.5)	0.124
**SUVpeak**	8.3 (1.3-30.1)	8.2 (2.4-24.7)	0.943	8.7 (3.1-30.1)	6.4 (1.3-20.1)	9.4 (3.6-23.2)	0.089
**SUVmean**	5.3 (1.6-21.5)	5.4 (1.9-14.9)	0.874	5.9 (1.9-21.5)	4.2 (1.6-14.5)	6.5 (2.8-20.3)	0.889
**MTV**	16.8 (0.5-313.3)	15.5 (1.5-118.8)	0.984	16.7 (0.9-118.3)	14.2 (0.5-121.0)	21.8 (1.5-313.3)	0.417
**TLG**	26.1 (0.7-1768.5)	25. 9(5.1-900.1)	0.915	28.7 (3.3-900.1)	21.7 (0.7-1768.5)	25.4 (2.9-70.5)	0.853

1 ^18^F-FDG PET/CT = ^18^F-fluorodeoxyglucose positron emission tomography/computed tomography; EAC = esophageal adenocarcinoma; ESCC = esophageal squamous cell carcinoma; MTV = metabolic tumor volume; SUV = standardized uptake value; TLG = total lesion glycolysis; Th1 = therapeutic group 1 (surgical resection only); Th2 = therapeutic group 2 (surgical resection plus chemotherapy and/or radiotherapy); Th3 = therapeutic group 3 (chemotherapy and/or radiotherapy without prior surgical resection).

1 Continuous variables are presented as median (range). Differences between histopathological groups (EAC and ESCC) were assessed using the Mann-Whitney U test. Differences between therapeutic groups (Th1–Th3) were assessed using the Kruskal-Wallis test.

Table 4 shows the most common metastatic sites in patients classified into the G2 and G3 groups, based on the metabolically most active lesion detected on ^18^F-FDG PET/CT.

**TABLE 4.** Distribution of metastatic sites in G2 and G3 groups

Site of metastasis	G2 (n=34)	G3 (n=33)
**Supradiaphragmatic lymph node**	11	10
**Lungs/pleura**	8	3
**Abdominal solid organs**	6	6
**Infradiaphragmatic lymph nodes**	3	6
**Bones**	5	5
**Small intenstine/large intestine**	1	2
**Peritoneum**	-	1

1 ^18^F-FDG PET/CT = ^18^F-fluorodeoxyglucose positron emission tomography/computed tomography; G2 = metastases without locoregional recurrence; G3 = locoregional disease and metastases

### Follow-up

Of the total 77 patients initially included in the study, 72 completed all study phases and were included in the final analysis. Median follow-up for the cohort was 70 months (95% CI: 26–105 months), estimated using the reverse Kaplan–Meier method. Median PFS was 10 months (95% CI: 9–14 months). Median OS was 27 months (95% CI: 15–40 months). Progression of disease was present in 55/72 (76.4%). Death was present in 43/72 (59.7%).

In this phase of the study, the final analysis consisted of 10 subjects in group G1, 32 subjects in group G2, and 30 subjects in group G3. The frequency of disease progression and death in each group was analyzed individually, and then their mutual comparison was made. A statistically significant difference was observed in the frequency of disease progression, which was significantly higher in groups G2 and G3 compared to group G1 (p = 0.001). On the other hand, there was no statistically significant difference in the frequency of death among the observed groups (P = 0.112). At the same time, the medians for PFS and OS were calculated and compared for each group individually, whereby no statistically significant difference was observed in median values for either PFS (p = 0.190) or OS (p = 0.707) (Table 5).

**TABLE 5.** Clinical outcomes according to ^18^F-FDG PET/CT groups

Outcome	G1	G2	G3	P
**N**	10	32	30	-
**Progression** Yes/No	3/7	27/5	25/5	**0.001**
**Mortality** Yes/No	3/7	20/12	20/10	0.112
**PFS median (range)**	18.0 (7-105)	11.0 (6-125)	10.0 (6-116)	0.190
**OS median (range)**	21.0 (10-105)	18.5 (6-132)	15.5 (6-126)	0.707

1 ^18^F-FDG PET/CT = ^18^F-fluorodeoxyglucose positron emission tomography/computed tomography; G1 = isolated locoregional disease; G2 = metastases without locoregional recurrence; G3 = locoregional disease and metastases; OS = overall survival; PFS = progression-free survival

1 P values were calculated using the Chi-square test for categorical variables. Continuous variables (PFS and OS) were analyzed using the Kruskal-Wallis test. Pairwise post hoc comparisons are not presented when the overall P value was not statistically significant.

### Association of ^18^F-FDG PET/CT parameters with survival outcomes

**FIGURE 2.**
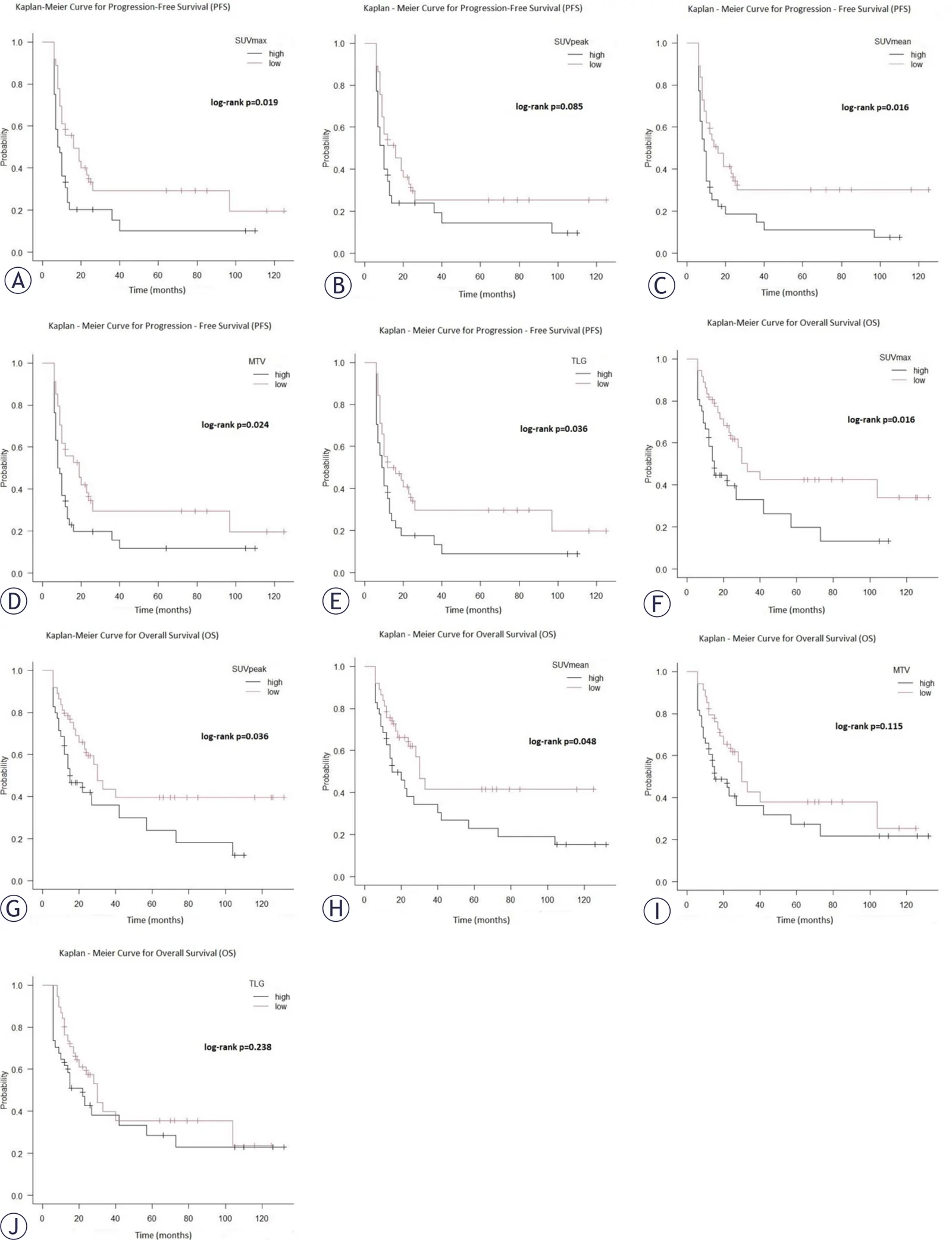
Kaplan–Meier survival curves showing progression-free survival **(A-E)** and overall survival **(F-J)** according to the median values of SUVmax, SUVpeak, SUVmean, metabolic tumor volume (MTV), and total lesion glycolysis (TLG) (high *vs*. low). The median values used as cut-offs for stratification were: SUVmax = [9.9], SUVpeak = [8.2], SUVmean = [5.4], MTV = [16.5], and TLG = [25.9].

Higher values of SUVmax, SUVmean, MTV, and TLG were associated with significantly shorter median PFS (p = 0.019, p = 0.016, p = 0.024, and p = 0.036, respectively). Although a difference in median PFS was observed for SUVpeak, it did not reach statistical significance (p = 0.085) (Figures 2A-E) (Table 6).

**TABLE 6.** Median progression-free and overall survival times according to low and high ^18^F-FDG PET/CT parameters

Parameter	Cut-off (median)	Median PFS Low vs. high (months)	Median OS Low vs. high (months)
**SUVmax**	9.9	8.5 vs. 16.0	15.0 vs. 33.0
**SUVpeak**	8.2	10.0 vs. 16.0	15.0 vs. 30.0
**SUVmean**	5.4	9.0 vs. 16.0	15.0 vs. 30.0
**MTV**	16.5	8.5 vs. 19.0	15.0 vs. 30.0
**TLG**	25.9	9.5 vs. 14.0	22.0 vs. 30.0

1 ^18^F-FDG PET/CT = ^18^F-fluorodeoxyglucose positron emission tomography/computed tomography; MTV = metabolic tumor volume; OS = overall survival; PFS = progression-free survival; SUV = standardized uptake value; TLG = total lesion glycolysis

Higher values of SUVmax, SUVpeak, and SUVmean were associated with shorter median OS (p = 0.016, p = 0.036, and p = 0.048, respectively). In contrast, MTV and TLG did not show a statistically significant association with OS (p = 0.115 and p = 0.238, respectively) (Figures 2F-J) (Table 6).

## Discussion

The main innovation of our study is reflected in a specific approach to the classification of patients with suspected recurrence esophageal carcinoma based on ^18^F-FDG PET/CT findings, which differs from the approaches used in previous studies. To our knowledge, studies that specifically stratified patients according to isolated locoregional recurrence, isolated metastases, and the simultaneous presence of locoregional recurrence and metastases on ^18^F-FDG PET/CT are still rare. In our study, patients with positive ^18^F-FDG PET/CT findings in the form of metastases or a combination of metastases and local recurrence (groups G2 and G3) significantly more frequently had elevated CEA blood levels compared to patients with isolated locoregional findings (G1). This result is in accordance with literature data showing that high serum CEA levels in esophageal carcinoma are more frequently associated with the presence of metastases.^11,12^

On the other hand, no statistically significant difference in the frequency of elevated CA19-9 levels was observed among the groups. Das *et al*., in a study that included patients with ESCC who had not previously undergone treatment, demonstrated that CA19-9 had higher sensitivity compared to CEA.^13^ In contrast, Ansari *et al*. reported that CEA blood levels have higher sensitivity than CA19-9.^14^ It is important to note that the participants in our study were more similar to the patients included in the study by Ansari *et al*., as that cohort consisted of post-treatment patients, including both EAC and ESCC cases. These results may suggest that CA19-9 has greater diagnostic value in patients before any treatment, while CEA may be more important for identifying and monitoring esophageal carcinoma recurrence after treatment.

Our results showed that disease progression was significantly more frequent in groups G2 and G3 compared to group G1. This finding may be explained by the fact that patients in group G1 had isolated locoregional recurrence, which potentially allows for better disease control through the applied therapeutic modalities and a lower chance of disease progression.^15,16^ In our study, ^18^F-FDG PET/CT parameters of the metastatic lesion with the highest metabolic activity were used, reflecting the metabolic characteristics of the biologically most aggressive tumor lesion. Accordingly, group G3 showed the highest values for all ^18^F-FDG PET/CT parameters evaluated. Increased glycolytic activity, accompanied by high values of ^18^F-FDG PET/CT parameters, was more often reported in more aggressive forms of the disease and advanced stages of malignancy.^17^

In our investigation, SUVmean demonstrated the highest statistical significance (p < 0.001) among all analyzed semiquantitative and quantitative ^18^F-FDG PET/CT parameters when comparing the three groups. This observation may be explained by the fact that SUVmean reflects the average metabolic activity across the tumor lesion, unlike SUVmax, which represents only the single voxel with the highest ^18^F-FDG uptake within the tumor.^18^

Semiquantitative and quantitative ^18^F-FDG PET/CT parameters were similar in patients with EAC and ESCC, with no statistically significant differences in median values according to histopathological tumor type. In previous studies analyzing these parameters in primary esophageal carcinoma before therapy, ESCC generally showed higher values.^19,20^ On the other hand, comparison of our results with the study by Korkmaz *et al*., which included patients after therapy, revealed a substantial similarity in findings.^21^

Progression-free survival and OS were shortest in patients from the G3 group; however, these differences did not reach statistical significance. Similarly, Kahraman *et al*. also stratified patients into three groups according to ^18^F-FDG PET/CT findings and evaluated differences in OS.^9^ Nevertheless, their classification was not entirely comparable to ours. In their study, OS was longest in patients with isolated locoregional disease and shortest in those with metastatic involvement, with statistically significant differences between the groups. Although our findings demonstrate a similar trend, the lack of statistical significance in our study may be attributed to differences in patient stratification criteria, sample size and the potential heterogeneity of the study population.

Our patients, who were classified into the “low” group according to SUVmax and SUVmean values, demonstrated statistically significantly longer PFS as well as OS. Patients with lower values of volumetric parameters (MTV and TLG) also had a statistically significantly higher median PFS. It is interesting that patients with lower values of volumetric parameters had longer OS, but without statistical significance compared to patients with higher values of these parameters. These results indicate that volumetric parameters better reflect the biological dynamics of the disease and the probability of earlier progression, while overall survival is additionally influenced by numerous other factors, including the therapeutic approach, the general condition in the patient, and the presence of comorbidities.

The prognostic significance of MTV and TLG parameters remains controversial in the literature, as some studies have demonstrated a significant association with survival outcomes, whereas others have failed to confirm such results.^22,23^ These discrepancies may be attributed to heterogeneity among study populations, differences in disease stage and therapeutic approaches, as well as methodological variations between studies, population heterogeneity, and a variable number of subjects.

However, the results of this study should be interpreted in the context of certain limitations. The study had a retrospective design and was conducted in one center, with a relatively small number of subjects. Additionally, the number of patients was not evenly distributed between all three groups, which could affect the comparison of the parameters analyzed among them.

Despite the mentioned limitations, our results provide a new approach to the classification of patients with esophageal carcinoma based on ^18^F-FDG PET/CT findings and open the possibility for further research into the prognostic significance of different disease patterns on ^18^F-FDG PET/CT. Based on the obtained results, it may be worth considering whether the standard classifications of esophageal cancer recurrence should be supplemented by this or a similar classification, and whether its potential value could be assessed through comparison with existing systems in future studies. Future prospective multicenter studies with a larger number of subjects are necessary to confirm the obtained findings.

## Conclusions

The main innovation of our study is reflected in a specific approach to the classification of patients with suspected recurrence esophageal carcinoma based on ^18^F-FDG PET/CT findings, which differs from the approaches used in previous studies. To our knowledge, studies that specifically stratified patients according to isolated locoregional recurrence, isolated metastases, and the simultaneous presence of locoregional recurrence and metastases on ^18^F-FDG PET/CT are still rare. The results of our study indicate that the pattern of disease spread on ^18^F-FDG PET/CT, in combination with semiquantitative and quantitative ^18^F-FDG PET/CT parameters, may have significant prognostic value in patients with esophageal carcinoma. Patients with simultaneous locoregional recurrence and metastases are particularly distinguished, in whom the highest values of ^18^F-FDG PET/CT parameters, more frequent disease progression, and the shortest survival were registered.
